# Application of Ionic Liquid Crosslinked Hydrogel for Removing Heavy Metal Ions from Water: Different Concentration Ranges with Different Adsorption Mechanisms

**DOI:** 10.3390/polym15132784

**Published:** 2023-06-22

**Authors:** Jian Sun, Ziqi Jin, Jiyang Wang, Hong Wang, Qian Zhang, Huajing Gao, Zhaohui Jin, Jianlin Zhang, Zhiwei Wang

**Affiliations:** 1 Institute of Petrochemical Technology, Jilin Institute of Chemical Technology, Jilin 132022, China; sunjian@jlict.edu.cn (J.S.);; 2 Shandong Chambroad HoldingGroup Co., Ltd., Binzhou 256599, China; 3 Shandong Efirm Biochemistry and Environmental Protection Co., Ltd., Binzhou 256500, China; 4 Key Laboratory of Clean Pulp & Papermaking and Pollution Control of Guangxi, College of Light Industrial and Food Engineering, Guangxi University, Nanning 530004, China

**Keywords:** heavy metal ions, hydrogels, ionic liquid, crosslinking agent

## Abstract

Heavy metal wastewater poses a significant environmental challenge due to its harmful effect on organisms and difficult biodegradation. To address this issue, hydrogel has been used as a promising solution for the adsorption of heavy metal ions in water, offering advantages such as low cost, simple design, and environmental friendliness. In this study, we synthetized a novel poly-acrylamide/acrylic acid/vinyl imidazole bromide (PAM/AA/[Vim]Br_2_) hydrogel as an effective adsorbent for the removal of Ni^II^, Cu^II^, Zn^II^, and Cr^III^ from water. The structure of the hydrogel was characterized by using techniques such as Fourier transform infrared spectroscopy (FTIR) and scanning electron microscopy (SEM). By exploring various parameters such as monomer ratio, neutralization degree, crosslinking agent addition amount, and initiator addition amount, the highest swelling ratio of the PAM/AA/[Vim]Br_2_ hydrogel reached 40,012%. One of the notable aspects of this study lay in the investigation of the adsorption behavior of the hydrogel towards heavy metal ions at different concentrations. The adsorption isotherm calculations and X-ray photoelectron spectroscopy (XPS) analysis revealed distinct adsorption mechanisms. At low concentrations, the hydrogel exhibits a multilayer physical adsorption mechanism, with heavy metal ion removal rates exceeding 80%; while at high concentrations, it demonstrates a monolayer chemical adsorption mechanism, with heavy metal ion removal rates above 90%. This dual mechanism approach distinguishes our study from previous reports on the removal of heavy metal ions using hydrogels and shows good ion adsorption efficiency at both high and low concentrations. To the best of our knowledge, this is the first report to explore the removal of heavy metal ions from water using hydrogels with such intriguing dual mechanisms. Overall, the utilization of the PAM/PAA/[Vim]Br_2_ hydrogel as an adsorbent for heavy metal ion removal presents a promising and innovative approach, contributing to the development of environmentally friendly solutions for heavy metal wastewater treatment.

## 1. Introduction

With the rapid development of industries, the problem of water pollution has become increasingly severe [1,2,3,4]. Among the various types of water pollutants, heavy metal ion pollution poses significant threats to ecosystems, human health, production, and the daily life of individuals [5,6,7]. Currently, several main treatment methods for removing heavy metal ions from water are employed, including filtration [8], ion exchange [9], oxidation–reduction [10,11], and chemical precipitation [12]. However, these treatment methods have inherent limitations, such as complicating the composition of wastewater, low removal efficiency, high costs, generation of large amounts of sludge, and potential secondary pollution. Activated carbon, a widely used porous material for water treatment through adsorption [13,14], has certain limitations that restrict its broad application, such as high experimental costs, difficulties in separation, and long adsorption time. Therefore, there is a need to develop environmentally friendly adsorbents with high removal efficiency, simple structural design, and low cost to address the treatment of wastewater containing heavy metal ions.

Hydrogels possess an extremely hydrophilic three-dimensional network structure, which can rapidly swell in water and retain a large volume of water without dissolving [15,16,17,18]. Due to their structural design, low cost, good water permeability, and biodegradability, hydrogels find wide applications in adsorbing heavy metal ions from wastewater [19,20,21,22,23]. Zahra [24] synthesized magnetic hydrogel beads based on poly(vinyl alcohol)/carboxymethyl starch-g-poly(vinylimidazole) for the removal of CuII and CdII, achieving removal rates (RR%) of 93.2% and 62.5%, respectively. The hydrogel exhibited a high RR (%) for heavy metal ions at a low concentration of 20 ppm. However, as the concentration of metal ions increased, the RR (%) gradually decreased. Shah [25] synthesized a PAA/PAM superabsorbent polymer hydrogel for the removal of CdII, NiII, and CuII from aqueous samples. The RR (%) for CdII, NiII, and CuII exceeded 75% across the entire concentration range. When the metal ion concentration was high (100 ppm), the hydrogel showed a high RR (%); however, the RR (%) was extremely low at low concentrations. Previous studies have also reported that chitosan/polyethyleneimine hydrogels exhibited removal rates were lower than 75% for Pb^2+^, Ni^2+^, and Cu^2+^ after multiple cycles of use at high concentration (100 ppm) [26], and the composite hydrogels exhibited a removal efficiency of 80% for Cu^2+^ at a concentration of 100 ppm [27]. Although these hydrogels demonstrated high removal efficiency for heavy metal ion adsorption at low or high concentrations, there have been no reports of hydrogels exhibiting high RR for heavy metal ions in both low and high concentration ranges.

Ionic liquids, also known as low-temperature molten salts, are a class of compounds composed entirely of ions and exhibit a liquid state at room temperature. Different from traditional high-temperature molten salt such as NaCl, due to the good symmetry of anion and cation and small ion radius, they can be firmly combined by electrostatic force [28,29,30]. In addition, ionic liquids show great potential in catalysis, separation, and electrochemistry due to their high thermal stability, wide electrochemical window, and structural designability [31,32,33,34,35]. However, the use of ionic liquids as crosslinking agents of hydrogels in the synthesis for adsorption of heavy metal ions in water has not been reported.

In this work, we use ionic liquid [Vim]Br_2_ as a crosslinking agent to synthesize PAM/AA/[Vim]Br_2_ hydrogel, which was mainly used to adsorb heavy metal ions such as Ni^II^, Cu^II^, Zn^II^, and Cr^III^ from water. The optimal conditions for hydrogel synthesis were optimized by response surface methodology. The swelling ratio of PAM/AA/[Vim]Br2 hydrogel could reach 40,012%. The effects of temperature, pH, initial concentration of heavy metal ions, and the amount of hydrogel added are discussed. The adsorption isotherm and XPS analysis indicated that the hydrogel exhibits different adsorption mechanisms when adsorbing heavy metal ions with different concentrations. At low concentrations, the adsorption of the hydrogel was attributed to multilayer physical adsorption, and the enhanced removal rates of heavy metal ions could be attributed to the electrostatic interaction of the bromide ions from the crosslinking ionic liquid agent in the hydrogel structure. On the one hand, this is the first report of achieving removal rates exceeding 80% at low concentrations using hydrogels. On the other hand, at high concentrations, the adsorption of the hydrogel follows a monolayer chemical adsorption mechanism, and the removal rates of heavy metal ions above 90% could be attributed to the chelation interaction between the active sites and the metal ions.

## 2. Experimental Methods

### 2.1. Materials

Acrylic acid (AA), acrylamide (AM), ammonium persulfate (APS), 1-vinylimidazole (Vim), 1,2-dibromoethane, nickel(II) chloridehexahydrate (NiCl_2_·6H_2_O), copper(II) chloride hexahydrate (CuCl_2_·6H_2_O), zinc(II) acetate dihydrate (Zn[CH_3_COO]_2_·2H_2_O), chromium(III) chloride hexahydrate (CrCl_3_·6H_2_O), sodium hydroxide (NaOH), ether, and methanol were purchased from Energy-Chemical (Shanghai, China). All chemicals were of analytical grade and used without any purification. The experimental water was ultrapure water prepared by an ELGA CLXXDM2 ultrapure water instrument (≥18.2 MΩ·cm).

### 2.2. Synthesis of [Vim]Br_2_

**Scheme 1:**
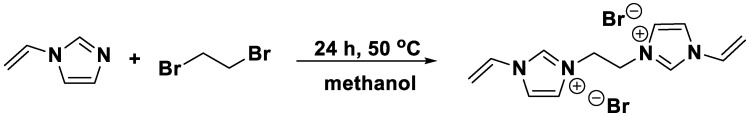
The synthesis of [Vim]Br_2_.

Under N_2_ protection, 1,2-dibromoethane (1.88 g, 10 mmol) was dissolved in 10 mL methanol, meanwhile, 1-vinylimidazole (1.88 g, 20 mmol) was dripped. The reaction mixture was stirred and heated to 50 °C for 24 h. The methanol solution was removed by vacuum distillation and yellow powder product was obtained. The crude product was washed 3 times with ether and dried under vacuum at 50 °C for 2 h. Finally, the yellow powdery product was obtained (Scheme 1).

The yield of Bis1-vinylimidazole ethyl bromide ([Vim]Br_2_) was 89%. ^1^HNMR (400 MHz, D_2_O), δ:4.8 (m, 4H), 5.43 (m, 2H), 5.78 (m, 2H), 7.1 (m, 2H), 7.56(m, 2H), 7.8 (m, 2H), 9.11 (s, 2H).

### 2.3. Preparation of PAM/AA/[Vim]Br_2_ Hydrogel

**Figure 1:**
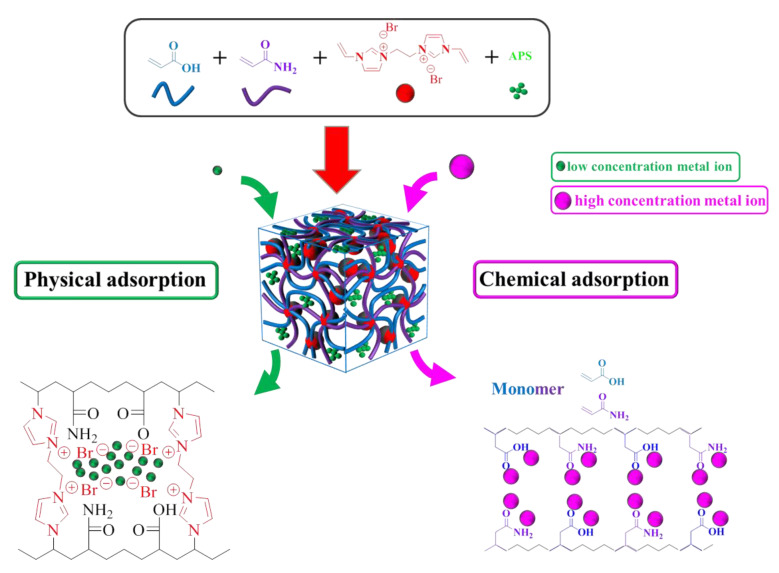
The synthetic process of hydrogel and its adsorption mechanisms for heavy metal ions.

Appropriate amounts of acrylic acid (AA), acrylamide (AM), NaOH, and distilled water were mixed. The mixture was stirred and cooled to room temperature. Then, a certain amount of crosslinking agent [Vim]Br_2_ was added and stirred magnetically at room temperature for 30 min. N_2_ was introduced to drain the oxygen in the flask and APS was added to seal the mixture. The polymerization was carried out in a water bath at 60 °C for 6 h. The hydrogels were washed with distilled water several times to remove unreactive monomers, dried at 60 °C until constant weight, crushed, screened, and reserved. Figure 1 shows the synthetic process of hydrogel and its adsorption mechanisms for heavy metal ions.

### 2.4. Hydrogel Swelling

The swelling behavior of PAM/AA/[Vim]Br_2_ hydrogels was investigated by immersion of 0.1 g of the SPH in 100 mL Milli-Q distilled water at room temperature and the hydrogels reached equilibrium swelling for 12 h. The influence of pH on the swelling behavior was tested using HCl and NaOH. Equation (1) calculates the percentage of hydration.

(1) $$\mathrm{Swelling}=\frac{m_{w}{-m}_{d}}{m_{d}}\times 100\%\;$$

where m_w_ is the mass of the swollen sample at time t and m_d_ is the weight of the dry sample.

### 2.5. Adsorption Experiments

First, 0.1 g of dry hydrogel powder was added to a 200 mL Erlenmeyer flask, 100 mL of heavy metal solutions (Ni^II^, Cu^II^, Zn^II^, Cr^III^) was added at different concentrations, and it was shaken at 25 °C for 12 h. After reaching the adsorption equilibrium, an atomic absorption spectrometer was used to detect the metal ion content in the remaining solution. Q_e_ and RR (%) were calculated using the following Equations (2) and (3):

(2) $${\;Q}_{e}=\frac{{(C}_{0}{-C}_{e})\times V}{M}$$

(3) $$\mathrm{RR}\%=\frac{C_{0}{-C}_{e}}{C_{0}}\times 100\%\;$$

Q_e_ (mg/g) represents the equilibrium removal efficiency of hydrogel. C_0_(mg/L) and C_e_ (mg/L) are the initial and the equilibrium concentrations of metal ions in liquid phase, respectively. V(L) is the volume of metal solution and M(g) is the weight of dried hydrogel.

### 2.6. Design of Response Surface Experiment

**Figure 2:**
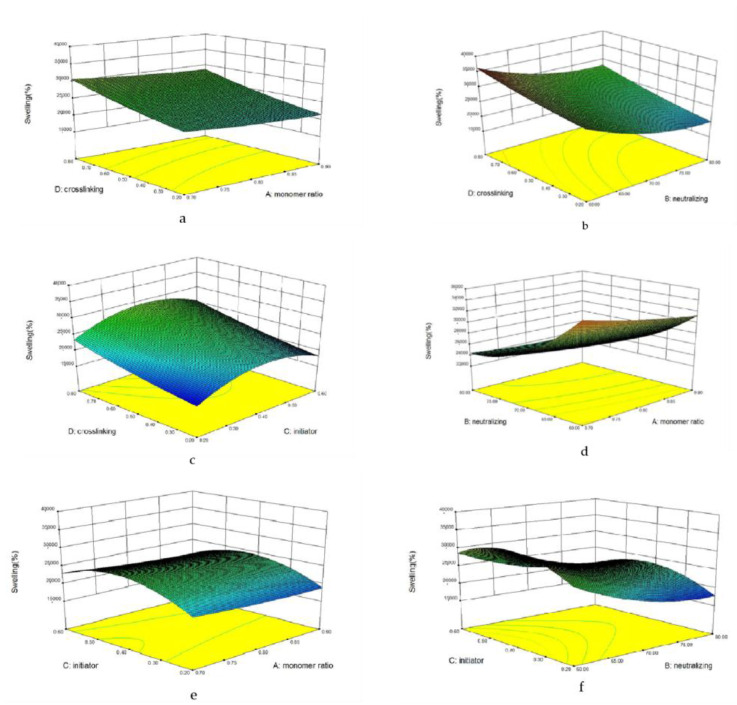
The influence of various factors on the response value in the response surface three-dimensional graph. The *z*-axis coordinate of six graphs is swelling (%). The horizontal and vertical coordinates are variables: (**a**) monomer ratio and crosslinking; (**b**) neutralizing and crosslinking; (**c**) initiator and crosslinking; (**d**) monomer ratio and neutralizing; (**e**) monomer ratio and initiator; (**f**) crosslinking and initiator.

The response surface method was used to design the experiment, and the factors affecting the swelling performance of the hydrogel were evaluated. There are four main variables: monomer ratio (A, 0.7–0.9%), neutralizing (B, 60–80), initiator (C, 0.2–0.6), and the crosslinking agent (D, 0.2–0.8). The analysis of variance of the results was carried out using the following quadratic model [36]:

$${Y=\beta }_{0}{+\beta }_{1}{A+\beta }_{2}{B+\beta }_{3}{C+\beta }_{4}{D+\beta }_{12}{\mathrm{AB}+\beta }_{13}{\mathrm{AC}+\beta }_{14}{\mathrm{AD}+\beta }_{23}\mathrm{BC}\;{+\beta }_{24}{\mathrm{BD}+\beta }_{34}{\mathrm{CD}+\beta }_{11}A^{2}{+\beta }_{22}B^{2}{+\beta }_{33}C^{2}{+\beta }_{44}D^{2}$$

where Y is the percentage of hydrogel swelling rate responses, β_n_ is the linear regression coefficients, A, B, C, and D are the studied factors (shown in Table 1). Values of “prob>F” which are less than 0.05 indicate that model terms are significant [37]. As shown in Figure 2, the response surface diagram shows that the swelling rate of the gelatin is affected by the interaction of four factors, rather than a single linear relationship. Optimization by four factors indicates: monomer ratio: 70:30, neutralizing: 60%, initiator: 0.4 wt%, crosslinking agent: 0.8 wt%, the maximum swelling rate of hydrogels reached 40,012%.

### 2.7. Characterization

^1^H NMR spectra were recorded at 400 MHz on a Bruker Ascend 400 spectrometer (Bruker Daltonics Inc., Billerica, MA, USA) with tetramethylsilane as the internal standard. The Fourier transform infrared (FTIR) spectroscopy was performed on a Nicolet iS5FTIR spectrometer equipped with an attenuated total reflectance (ATR) accessory. The samples were first mixed with dried KBr before analysis and the spectrum of each sample was obtained in the range of 4000–500 cm^−1^. The surface morphology of hydrogels was observed with a scanning electron microscope JSM-6490LV (JEOL, Tokyo, Japan). The XPS measurements were conducted on an ESCALAB 250Xi spectrometer. An atomic absorption spectrometer (AAS) was applied for the determination of the metal ions in the aqueous medium.

## 3. Results and Discussion

### 3.1. FTIR Analysis

**Figure 3:**
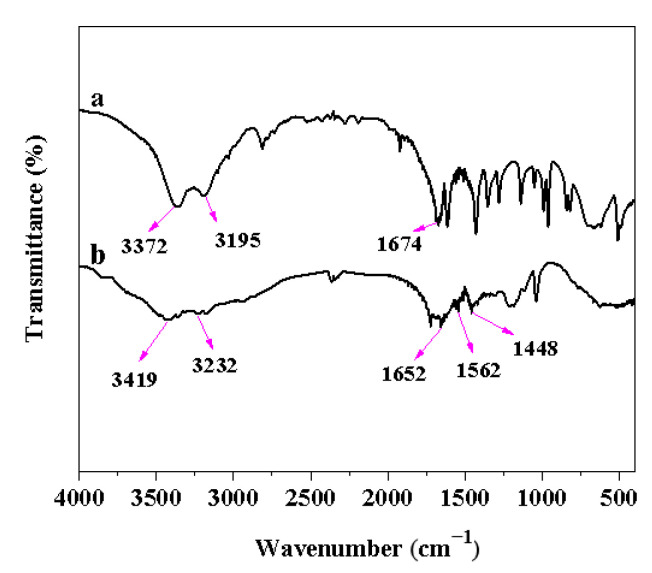
(**a**) Infrared spectrum of acrylamide (AM). (**b**) PAM/AA/[Vim]Br_2_ hydrogel.

In the infrared spectrum of AM (Figure 3a), the strong absorption bands of amide groups are observed at 3372 and 3195 cm^−1^. In the case of PAM/AA/[Vim]Br_2_ hydrogel (Figure 3b), the strong absorption peaks of amide groups have shifted to 3419 and 3232 cm^−1^. Additionally, the C=O stretching vibration absorption peak at 1674 cm^−1^ in the AM infrared spectrum corresponds to the C=O stretching vibration absorption peak at 1652 cm^−1^ in the PAM/AA/[Vim]Br_2_ hydrogel. The peaks observed at 1562 and 1448 cm^−1^ in the hydrogel correspond to the C=O stretching vibration peaks of the carboxyl anion (-COO**^-^**) in AA. These data indicate the successful copolymerization of AA and AM [38], resulting in the synthesis of PAM/AA/[Vim]Br_2_ hydrogel.

### 3.2. SEM Analysis of PAM/AA/[Vim]Br_2_ Hydrogel

**Figure 4:**
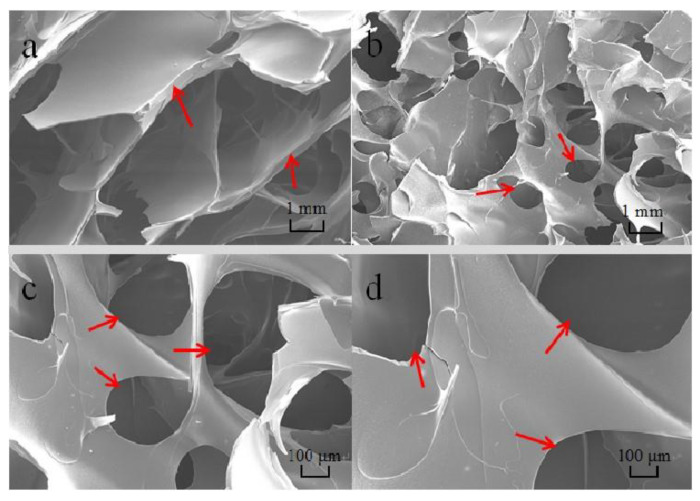
SEM images of PAM/AA/[Vim]Br_2_ hydrogel. (**a**,**b**) are 1 mm structural illustrations, (**c**,**d**) are 100 μm structural illustrations. The red arrows points to the internal pores of the hydrogel.

As shown in Figure 4a, after freezing intervention, the prepared PAM/AA/[Vim]Br_2_ hydrogel has a clearer layered structure, indicating that the internal molecular structure of the prepared hydrogel is more evenly distributed. As shown in Figure 4b, the internal pore size of the hydrogel is uniformly distributed, and it is a polymer material with a three-dimensional network structure. As shown in Figure 4c,d, the hydrogel has a large pore structure. This large loose pore structure further increases the contact area between the hydrogel and heavy metal ions, which is conducive to hydrogel adsorption of heavy metal ions.

### 3.3. Effect of Initial Concentration of Ni^II^, Cu^II^, Zn^II^, Cr^III^ on RR (%)

**Figure 5:**
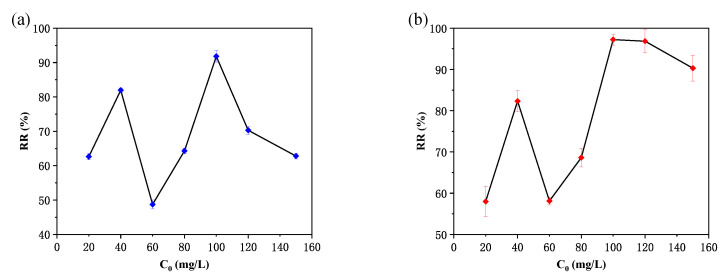

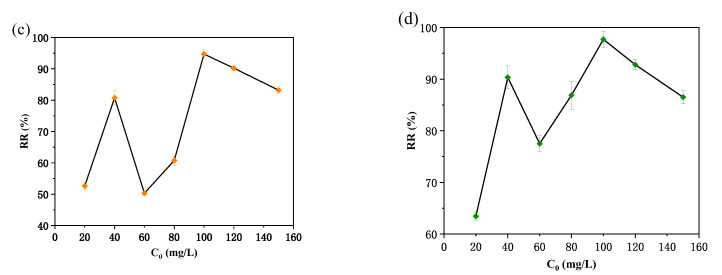
(**a**) Effect of initial concentration of Ni^II^ on RR (%). (**b**) Cu^II^ RR (%). (**c**) Zn^II^ RR (%). (**d**) Cr^III^ RR (%).

The effects of the initial concentrations of Ni^II^, Cu^II^, Zn^II^, and Cr^III^ on the removal rate (RR%) are depicted in Figure 5. Heavy metal solutions with initial concentrations of 20, 40, 60, 80, 100, 120, and 150 mg/L were selected for the experimental study. The four adsorption curves exhibited a consistent pattern and could be divided into two stages. In the first stage, within the low concentration range (<40 ppm), the adsorption capacities of the hydrogels for heavy metal ions increased as the initial concentration of metal ions rose. This could be attributed to the electrostatic attraction between anions, such as Br- and Ni^II^, towards heavy metal ions. However, as the metal ion concentration continued to increase (<60 ppm), the electrostatic force weakened, resulting in a decline in the removal rate. In the second stage, within the high concentration range (<100 ppm), the RR% of heavy metal ions increased as the concentration of metal ions rose. This could be attributed to the increased contact probability between the active sites in the hydrogel and the heavy metal ions, enhancing the coordination between the functional groups of the hydrogel and the metal ions. However, once the concentration surpassed a threshold value (>100 ppm), the adsorption capacity of the hydrogel approached saturation, and the RR% started to decrease. This decrease was due to the saturation of active sites on the hydrogel, which limited the coordination between the functional groups in the hydrogel and the metal ions. Given the generally consistent trends of the four metal ions in both the low and high concentration ranges, our subsequent study primarily focused on Ni^II^.

### 3.4. Effect of Hydrogel Dosage on RR (%)

**Figure 6:**
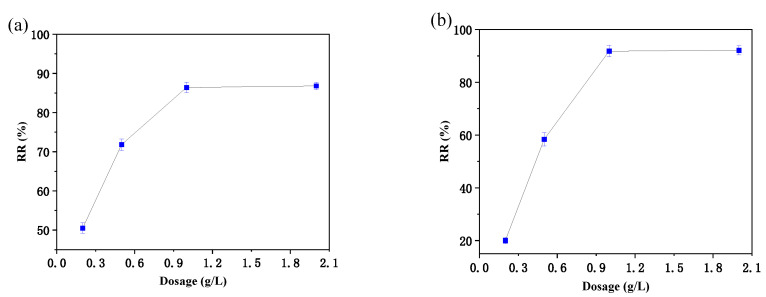
(**a**) Effect of hydrogel addition RR (%) (40 ppm); (**b**) 100 ppm.

The effect of the added amount of hydrogel on RR (%) is shown in Figure 6. The results showed that at low concentration (40 ppm) or high concentration (100 ppm), RR (%) increased sharply with the increase in hydrogel dosage, which mainly increased the surface area and active sites of hydrogel. When the hydrogel dosage was 2 g/L, the RR (%) value reached 86.4% at a low concentration. When the concentration was high, the RR (%) value reached 91.8%. However, when the hydrogel dosage was 1 g/L, the saturation phenomenon appeared, and the RR (%) increased slowly with the increase in hydrogel addition. Therefore, 1 g/L was selected as the best dosage for further adsorption experiments.

### 3.5. Effect of Temperature on RR (%)

**Figure 7:**
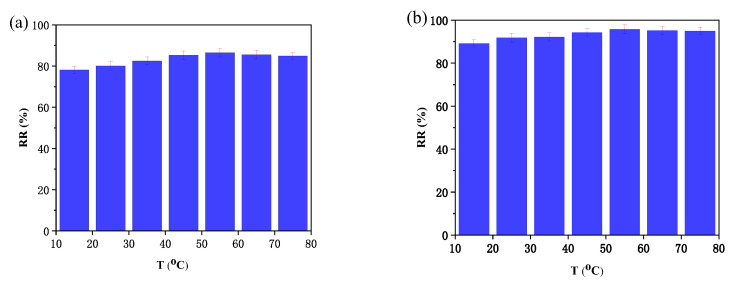
(**a**) Effect of temperature on RR (%) (40 ppm); (**b**) 100 ppm.

The changes in the adsorption capacity of the hydrogel for heavy ions at different temperatures are shown in Figure 7. As the temperature increased from 15 °C to 55 °C, the hydrogel’s removal rates (RRs) significantly increased. When the temperature exceeded 55 °C, there was little alteration observed in the removal efficacy for heavy metal ions. This phenomenon indicates that with increasing temperature, the activity of heavy metal ions is enhanced, leading to the disruption of hydrogen bonds between the hydrogel and water. This exposes more active functional groups for complexation with heavy metal ions [38]. However, when the adsorption capacity approaches saturation, further increasing the temperature does not significantly increase the removal efficiency of the hydrogel.

### 3.6. Effect of pH on RR (%)

**Figure 8:**
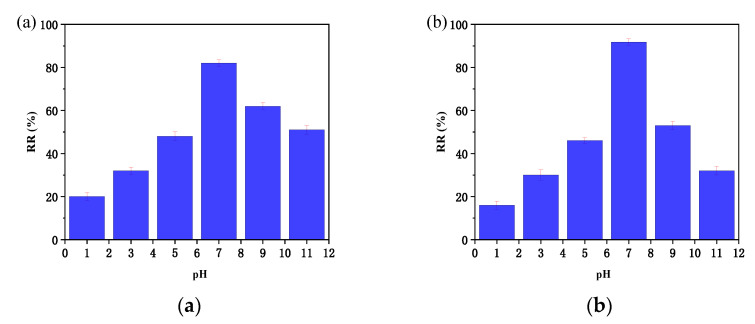
(**a**) Impact of pH on Ni^II^ adsorption onto hydrogel (40 ppm); (**b**) 100 ppm.

Figure 8 illustrated the RR (%) of PAM/PAA/[Vim]Br2 hydrogel (40 ppm, 100 ppm) for heavy metal ions (NiII, CuII, ZnII, CrIII) in solutions with different pH values. The RR (%) peaked at pH 7, while it significantly decreased in strong acid (pH = 3) or strong alkaline (pH = 11) environments compared to the neutral environment. This was because, in acidic conditions, H+ protonated the -COO^−^ and -NH_2_ groups on the hydrogel structure, forming -COOH and -NH_3_^+^. The H^+^ ions also competed with heavy metal ions for adsorption sites. As the solution pH gradually increased to 7, the competitive effect of H^+^ ions weakened, allowing a large number of -NH_2_ and -COO^−^ groups to re-coordinate with heavy metal ions, resulting in an increase in the RR (%) [39]. However, when the solution pH was above 7 and gradually increased to a strong alkaline environment, the increased OH^−^ ion concentration in the external solution led to an anion shielding effect on the -COO^−^ and -NH_2_ groups of the hydrogel structure. This caused a decrease in osmotic pressure inside and outside the hydrogel network, hindering the effective diffusion of heavy metal cations into the hydrogel. As a result, the RR (%) decreased [40].

### 3.7. Adsorption Kinetics of Heavy Metal Ions by PAM/AA/[Vim]Br_2_ Hydrogel

**Figure 9:**
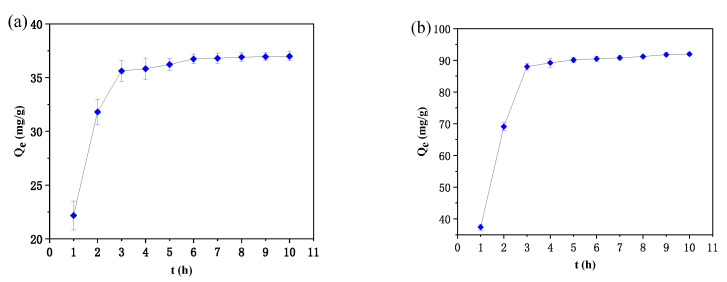
(**a**) Adsorption kinetics curves of PAM/AA/[Vim]Br_2_ hydrogels for heavy metal ions (40 ppm); (**b**) 100 ppm.

The adsorption effect of PAM/PAA/[Vim]Br2 hydrogel on Ni^II^ with initial concentrations of 40 ppm and 100 ppm at different time points (1, 2, 3, 4, 5, 6, 7, 8, 9, and 10 h) is shown in Figure 9. During the initial adsorption period (0–2 h), the adsorption of heavy metal ions by the hydrogel increased rapidly. After 3 h of adsorption, the efficiency of heavy metal ion adsorption gradually decreased. At 4 h, the adsorption capacity of the hydrogel for heavy metal ions approached saturation. These adsorption behaviors indicated that PAM/PAA/[Vim]Br_2_ hydrogel could achieve relatively fast and near-saturation adsorption of heavy metal ions. [41,42].

The adsorption kinetics of PAM/PAA/[Vim]Br_2_ hydrogel on Ni^II^ was fitted by a pseudo-first-order kinetics and two-stage kinetic Equations (4) and (5), and the kinetics of the adsorption reaction of PAM/AA/[Vim]Br_2_ hydrogel on Ni^II^ was obtained. The equation expression is as follows.

(4) $$\;\ln\left(Q_{e}{-Q}_{t}\right){=\mathrm{lnQ}}_{e}{-k}_{1}t\;$$

(5) $$\frac{t}{Q_{t}}=\frac{t}{Q_{e}}+\frac{1}{k_{2}Q_{e}^{2}}\;$$

where t is the adsorption time (min); Q_e_ and Q_t_ are, respectively, the adsorption capacity of PAM/AA/[Vim]Br_2_ hydrogel for heavy metal ions when adsorbed at equilibrium time and t (mg/g); K_1_ and K_2_ are quasi-one- and quasi-two-stage adsorption rate constants, respectively.

**Figure 10:**
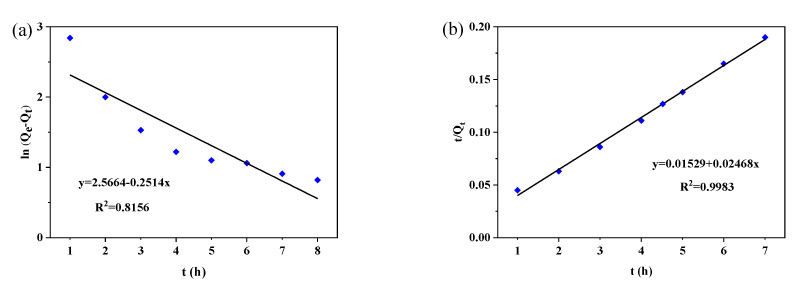

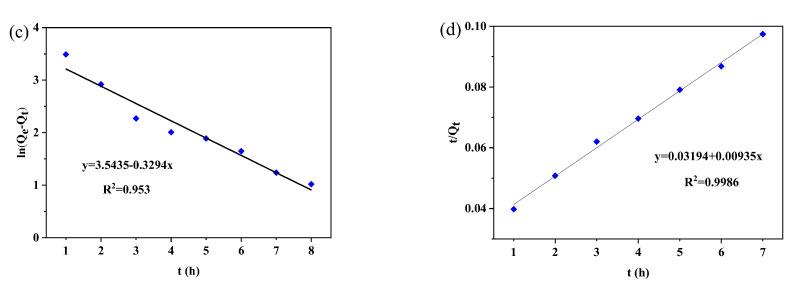
Fitting results of adsorption kinetics of Ni^II^ by PAM/AA/[Vim]Br_2_ hydrogel. (**a**,**b**) The first-order kinetics and second-order kinetics fitting results of 40 ppm, respectively; (**c**,**d**) 100 ppm.

The results of pseudo-first-order and pseudo-second-order reaction kinetics fitting curves of Ni^II^ at 40 ppm and 100 ppm are shown in Figure 10 and Table 2. The results show that: compared with the pseudo-first-order adsorption kinetics fitting results (40 ppm, R^2^ = 0.8156, Figure 10a; 100 ppm, R^2^ = 0.953, Figure 10c), the pseudo-second-order adsorption kinetic model fitting is more consistent (40 ppm, R^2^ = 0.9983, Figure 10b; 100 ppm, R^2^ = 0.9986, Figure 10d). Therefore, the adsorption of heavy metal ions by hydrogel (40 ppm, 100 ppm) is a multistep process. First, the heavy metal ions adhere to the metal surface and then enter the hydrogel through the channel of the hydrogel to further spread.

### 3.8. Adsorption Isotherms of PAM/AA/[Vim]Br_2_ Hydrogels for Heavy Metal Ions

The adsorption isotherms of Freundlich and Langmuir were studied. The Freundlich isotherm is a heterogeneous, multilayer adsorption system, and the absorption process takes place on an active heterogeneous surface. The Langmuir isotherm is a homogeneous, single molecular layer adsorption system, each binding site on the absorption surface absorbs the same energy, and each binding site is occupied by only one metal ion. The two models are presented in Equations (6) and (7):

(6) $${\mathrm{lnQ}}_{e}{=\mathrm{lnK}}_{F}+\frac{{\mathrm{lnC}}_{e}}{n}\;$$

(7) $$\frac{C_{e}}{Q_{e}}=\frac{1}{K_{L}Q_{m}}+\frac{C_{e}}{Q_{m}}\;$$

where C_e_, Q_e_, Q_m_ were the initial equilibrium concentration (mg/L) of heavy metal ion solution, the adsorption capacity of PAM/AA/[Vim]Br_2_ hydrogel to heavy metal ions (mg/g), and the saturated adsorption capacity of PAM/AA/[Vim]Br_2_ hydrogen to heavy metal ions (mg/g), K_F_ and K_L_ are Freundlich and Langmuir equilibrium constants, respectively, and n is the concentration index.

**Figure 11:**
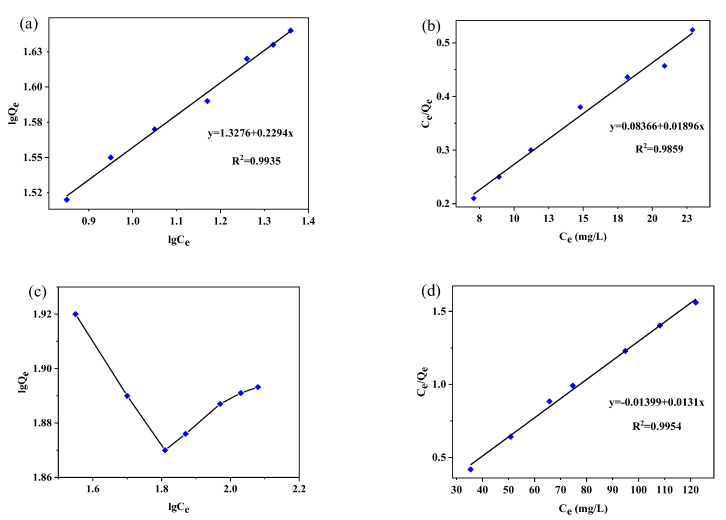
Fitting results of adsorption isotherms of Ni^II^ for hydrogel PAM/AA/[Vim]Br_2_ hydrogel. (**a**,**b**) The Freundlich adsorption isotherm and Langmuir adsorption isotherm fitting results of 40 ppm, respectively; (**c**,**d**) 100 ppm.

The Freundlich and Langmuir adsorption isotherm models were fitted to the initial concentration and equilibrium adsorption capacity of the PAM/AA/[Vim]Br_2_ hydrogel to adsorb Ni^II^. The results are shown in Figure 11 and Table 3. When the initial concentration of Ni^II^ was 40 ppm, regarding the adsorption isotherm of Ni^2+^ by the hydrogel, the Freundlich adsorption isotherm (R^2^ = 0.9935) (Figure 11a) is better than the Langmuir adsorption isotherm (R^2^ = 0.9859) (Figure 11b), which shows that the adsorption isotherm of hydrogel for Ni^II^ is more in line with multilayer physical adsorption. When the initial concentration of Ni^II^ is 100 ppm, regarding the adsorption isotherm of hydrogel for Ni^II^, the Langmuir adsorption isotherm (R^2^ = 0.9954) (Figure 11d) has better fitting results, which shows that the adsorption of Ni^II^ to hydrogel is more in line with multilayer chemical adsorption.

### 3.9. XPS Analysis of PAM/AA/[Vim]Br_2_ Hydrogel

**Figure 12:**
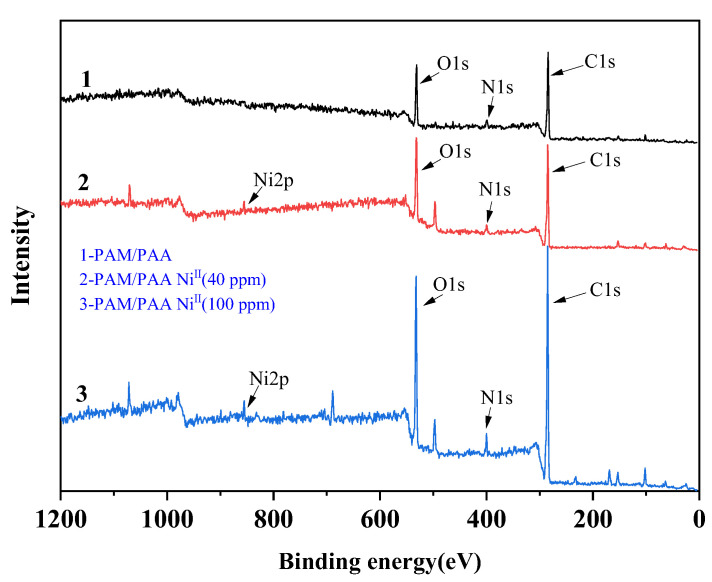
XPS spectrum of PAM/AA/[Vim]Br_2_ hydrogel before and after Ni^II^ ion adsorption.

**Figure 13:**
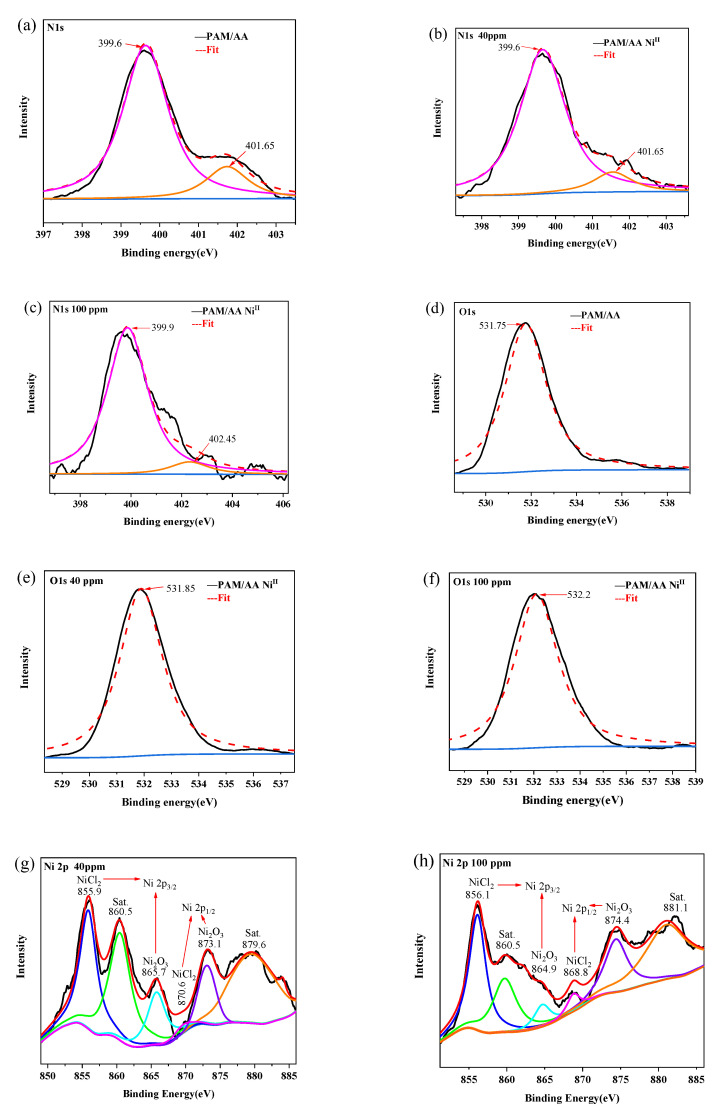
(**a**–**c**): XPS spectrum of N1s with PAM/AA hydrogel adsorbing NiII ions (0, 40, 100 ppm). (**d**–**f**): XPS spectrum of O1s with PAM/AA hydrogel adsorbing NiII ions (0, 40, 100 ppm). (**g**,**h**): XPS spectrum of Ni2p (40, 100 ppm) with a binding energy ranging from 849.1 eV to 886.2 eV (The black line represents the test line, the red line represents the fitting line, and other colors represent reference lines).

To further investigate the adsorption mechanism of hydrogels for heavy metal ions, XPS was used to analyze the binding energy of the hydrogel before and after adsorption, and the results are shown in Figure 12 and Figure 13.

In Figure 12, the main elements of the hydrogel such as the binding energy peaks of C1s, O1s, N1s can be seen. The Figure 12 (2) and (3) curves exhibit that the binding energy peak of Ni2p appears after adsorption, which proves that the Ni^II^ was adsorbed by the hydrogel. As shown in Figure 13a, in the PAM/AA sample before the adsorption of the Ni^II^ ions (40 ppm, 100 ppm), the N1s spectrum showed distinct peaks at ~399.6 eV and ~401.65 eV, corresponding to the -NH_2_ or -NH or C-NH^3+^ groups [43]. In the O1s range, the spectrum showed a distinct peak at ~531.75 (Figure 13d) [44,45], corresponding to the oxygen of C=O or C-O. After the adsorption of the Ni^II^ ions, the N1s spectrum shows no significant changes (40 ppm) (Figure 13b), however, the binding energy peaks of the N1s move from 399.65 eV to 399.95 eV and 401.65 eV to 402.45 eV (100 ppm) (Figure 13c). The O1s peak shifted slightly from 531.75 eV to 531.85 eV (40 ppm) (Figure 13e) and the O1s peak shifted strongly from 531.75 eV to 532.2 eV (100 ppm) (Figure 13f). In addition, Figure 13g (40 ppm) and Figure 13h (100 ppm) show the XPS spectrum of Ni2p with a binding energy ranging from 849.1 eV to 886.2 eV. Two major peaks with binding energies of 855.9 eV and 870.6 eV have a significant corresponding relationship to NiCl_2_, and the peaks of Ni_2_O_3_ are found at 865.7 eV and 873.1 eV (40 ppm) (864.9 and 874.4 (100 ppm)), which indicates that the PAM/PAA hydrogel can effectively offer O element as a chelating group for removal of Ni^II^ [46]. The other two peaks at 860.5 eV and 879.6 eV (40 ppm) (860.5 and 879.6 (100 ppm)) can be assigned to the corresponding satellite peaks of Ni 2p_3/2_ and Ni 2p_1/2_ [47]. The XPS spectra indicate that the concentration of heavy metal ions was low (40 ppm), and the adsorption of heavy metal ions by the hydrogel was mainly physical adsorption, which was the electrostatic attraction between ionic liquids and heavy metal ions. When the concentration of heavy metal ions was high (100 ppm), the adsorption of heavy metal ions by the hydrogel was mainly chemical adsorption, which was due to the chelation and coordination reaction of heavy metal ions with the amino, hydroxyl, and carboxyl groups of the hydrogel. The conclusion was consistent with the adsorption isotherm of hydrogel.

## 4. Conclusions

To our best knowledge, there have been no reports on the use of ionic liquids as crosslinking agents to prepare hydrogels for the adsorption of heavy metal ions in water. The experiment successfully synthesized a PAM/AA/[Vim]Br_2_ hydrogel using the ionic liquid [Vim]Br_2_ as the crosslinking agent, which was confirmed by FTIR characterization. In our experiment, under near-neutral solution conditions and at a heavy metal ion concentration of 100 ppm, the PAM/AA/[Vim]Br_2_ hydrogel demonstrated superior adsorption performance for Ni^II^, Cu^II^, Zn^II^, and Cr^III^, achieving the removal rates of 91.8%, 97.2%, 95.6%, and 98.1%, respectively, with RR% values all exceeding 90%. This indicates a certain advantage in heavy metal removal rates compared to the reported values. However, further testing in actual wastewater and assessment of the removal efficiency after multiple cycles of use are still required.

The adsorption isotherms and XPS analysis revealed that, at low concentrations (40 ppm), the hydrogel follows the Freundlich isotherm for adsorbing heavy metal ions, primarily through multilayer physical adsorption. One major highlight of this article is the removal rate of heavy metal ions in the low concentration range, which exceeds 80%. This is mainly attributed to the electrostatic interaction between anions in the ionic liquid and the heavy metal ions. At higher concentrations (100 ppm), the hydrogel follows the Langmuir isotherm, indicating monolayer chemical adsorption. This is mainly attributed to the coordination between carboxyl and amino groups within the hydrogel and the metal ions.

## Figures and Tables

**Table 1:** Independent variables and coded level of the experiment.

Variable	Code	Level		
Monomer ratio	A	0.7	0.8	0.9
% Neutralizing	B	60	70	80
% Initiator	C	0.2	0.4	0.6
% Crosslinking	D	0.2	0.5	0.8

**Table 2:** Fitting parameters of adsorption kinetics model of different concentrations of Ni^II^ for PAM/PAA/[Vim]Br_2_ hydrogel.

Ion	Concentration	Pseudo-First-Order			Pseudo-Second-Order		
		Q_e,1_ (mg/g)	K_1_	R^2^	Q_e,2_ (mg/g)	K_2_	R^2^
Ni^2+^	40 ppm	32.23	0.833	0.8156	38.28	0.01152	0.9983
	100 ppm	89.12	0.572	0.953	98.98	0.00418	0.9986

**Table 3:** Fitting parameters of adsorption isotherm model of different concentrations of Ni^II^ for PAM/PAA/[Vim]Br_2_ hydrogel.

Ion	Concentration	Langmuir			Freundlich		
		K_L_	Q_m_	R^2^	K_F_	n	R^2^
Ni^2+^	40 ppm	0.29	49.19	0.9859	816.58	3.33	0.9935
	100 ppm	−0.153	69.06	0.9954	86,377	−6.97	/

## Data Availability

The data presented in this study are available on request from the corresponding author.
